# Linking restless legs syndrome with Parkinson's disease: clinical, imaging and genetic evidence

**DOI:** 10.1186/2047-9158-1-6

**Published:** 2012-02-27

**Authors:** Tasneem Peeraully, Eng-King Tan

**Affiliations:** 1 Department of Neurology, Singapore General Hospital, Outram Road, Singapore 169608, Republic of Singapore

**Keywords:** Parkinson's disease, Restless-legs syndrome, Pathophysiology, Dopaminergic dysfunction.

## Abstract

Restless legs syndrome (RLS) and Parkinson's disease (PD) are both common neurological disorders. There has been much debate over whether an etiological link between these two diseases exists and whether they share a common pathophysiology. Evidence pointing towards a link includes response to dopaminergic agents in PD and RLS, suggestive of underlying dopamine dysfunction in both conditions. The extrastriatal dopaminergic system, in particular altered spinal dopaminergic modulation, may be variably involved in PD patients with RLS symptoms. In addition, there is now evidence that the nigrostriatal system, primarily involved in PD, is also affected in RLS. Furthermore, an association of RLS with the *parkin *mutation has been suggested. The prevalence of RLS has also been reported to be increased in other disorders of dopamine regulation. However, clinical association studies and functional imaging have produced mixed findings. Conflicting accounts of emergence of RLS and improvement in RLS symptoms after deep brain stimulation (DBS) also contribute to the uncertainty surrounding the issue. Among the strongest arguments against a common pathophysiology is the role of iron in RLS and PD. While elevated iron levels in the substantia nigra contribute to oxidative stress in PD, RLS is a disorder of relative iron deficiency, with symptoms responding to replacement therapy. Recent ultrasonography studies have suggested that, despite overlapping clinical features, the mechanisms underlying idiopathic RLS and RLS associated with PD may differ. In this review, we provide a concise summary of the clinical, imaging and genetic evidence exploring the link between RLS and PD.

## Introduction

The common features of dopaminergic dysfunction and response to dopaminergic agents in RLS and PD, together with comorbidity in some patients has fuelled the concept that these two diseases may share the same pathophysiology [1]. We review the clinical, imaging and genetic evidence, exploring the link between RLS (restless legs syndrome) and PD (Parkinson's disease).

### PD and RLS: an overview

PD is a progressive neurodegenerative disorder characterised by pathologic intraneuronal alpha-synuclein-positive Lewy bodies and neuronal cell loss. In particular, involvement of dopaminergic cells in the substantia nigra pars compacta is associated with development of the motor features of the disease. The cardinal clinical symptoms and signs of PD are bradykinesia, rigidity, tremor, postural instability and freezing attacks [2]. The prevalence of PD increases with age, affecting 1-2% of the population over the age of 65 years, and 3% of those over 85 years [3]. Several monogenic forms of PD and numerous genetic susceptibility factors have been identified [4,5]. Sleep disturbances have been widely reported in PD, although an increased incidence of periodic leg movements of sleep (PLMS) is debatable, with polysomnography studies revealing mixed findings [6-10].

Restless legs syndrome (RLS) was initially described by Ekbom [11] in 1945. It tends to present in mid to later life and has a prevalence of 2.7-10% in Western populations [12,13]. It is reportedly much less common amongst Asians [14]. Four essential diagnostic criteria were proposed by the International Restless Legs Syndrome Study Group (IRLSSG) in 1995; 1. An urge to move the legs associated with paresthesias, 2. motor restlessness, 3. worsening at rest with at least partial relief by activity, and 4. worsening at night [15,16]. In 2003, at a consensus conference at National Institute of Health, USA, these criteria were modified and new criteria developed for the diagnosis of RLS in the cognitively impaired elderly and in children [17]. Additional features supporting a diagnosis of RLS include sleep disturbance, periodic limb movement in sleep (PLMS), chronic symptoms with exacerbations and remissions, a positive family history and improvement of symptoms with dopaminergic drugs.

RLS can be associated with medical conditions such as renal failure, iron deficiency, neuropathy, and pregnancy [18-22]. Family history, with an autosomal dominant mode of inheritance may be present in more than half of the cases. Five genetic loci for RLS have been reported in different pedigrees, of which 4 are autosomal dominant and one autosomal recessive [23]. The detection of PLMS during overnight polysomnography is the the most frequent objective abnormality and can be demonstrated in the majority of RLS patients [24-27]. However, the absence of PLMS does not exclude RLS [26].

### Evidence of dopaminergic dysfunction in RLS

Observation studies of RLS in diseases which involve dopaminergic systems lend support to the hypothesis that dopaminergic dysfunction is present in RLS. One family with comorbidity of Huntington's disease (HD) and idiopathic RLS has been reported. All family members affected by RLS also had HD, but not vice versa [28]. Ondo and colleagues found a very high rate (33%) of undiagnosed RLS in their patients presenting with essential tremor. However, unlike other "secondary" forms of RLS, this finding was also associated with a high familial history of RLS [29]. A study of comorbidities in Tourette syndrome (TS) including RLS investigated 144 probands with TS or chronic tics and their parents. RLS was present in 10% of probands and 23% of parents with no gender differences [30].

Dopaminergic dysfunction has also been implicated in thyroid disorders. In a study of 146 patients with biochemically confirmed hyper- or hypothyroidism and 434 controls, none of the patients satisfied all the IRLSSG criteria of RLS [31]. However, 8.2% of patients had RLS-like symptoms (satisfying the first 3 IRLSSG criteria) compared to 0.9% of controls. Four (33.3%) of these patients reported complete resolution of their symptoms after their thyroid condition was treated. Interestingly, there was also a report of RLS emerging after the initiation of L-thyroxine treatment in a hypothyroid patient with low serum ferritin [32]. Upon withdrawal of L-thyroxine, there was a significant improvement in the RLS severity score, PML index, number of arousals due to PLMS, sleep efficiency, and biochemical parameters. Hence, RLS symptoms can complicate thyroxine replacement in hypothyroid patients with low serum ferritin.

### Pathological evidence of dopamine dysfunction in PD and RLS

Post-mortem studies in PD demonstrate loss of nigral neurons resulting in striatal dopamine deficiency, with differing morphological lesion patterns according to the clinical subtypes of PD. Cell loss in the ventrolateral part of the substantia nigra pars compacta (SNPC) projecting to the dorsal putamen is more prominent in the akinetic-rigid type, whereas tremor-dominant PD shows predominantly medial SNPC cell loss [33]. Variability in lesion patterning might explain why some patients with PD may develop RLS. In addition, loss of dopamine 2 (D2) receptors has been documented in advanced PD [33].

At autopsy of 8 patients with primary RLS, there was a significant decrease in dopamine 2 (D2) receptors in the putamen compared to a neurologically normal control group. The decrease in the D2 receptors correlated to the severity of the RLS [34]. This evidence that the nigrostriatal dopaminergic system is affected in both RLS and PD might provide a stronger argument for an etiologic link between the two. Moreover, there were significant increases in tyrosine hydroxylase in the substantia nigra, but not in the putamen of the RLS group. Phosphorylated (active) tyrosine hydroxylase was found to be increased in both the substantia nigra and putamen. These findings are consistent with data from animal iron deficiency models demonstrating increased presynaptic dopaminergic activity [34].

Ondo et al [35] performed bilateral stereotactic 6-hydroxydopamine (6-OHDA) lesions in dopaminergic diencephalic spinal (A11) neurons in rats resulting in a 54% reduction in A11 tyrosine hydroxylase staining cells compared to rats receiving sham treatment. Multiple 90- 120 minute video epochs demonstrated an increased average number of standing episodes and increased total standing time in four lesioned rats when compared with two sham rats. Treatment of the lesioned rats with intramuscular pramipexole subsequently resulted in fewer standing episodes and less total standing time. The results supported the authors' suspicion that RLS involves dopaminergic A11 cells.

Of note, post-mortem examinations in RLS patients have not demonstrated dopaminergic cell loss in SNPC [36], presence of Lewy bodies or positive alpha-synuclein immunohistochemistry staining [37].

### Genetic studies

*Parkin *mutations were found in 10 of 20 patients in 2 families with idiopathic RLS [38]. The clinical phenotype did not differ between RLS patients with and without a *parkin *mutation [38]. A recent study of 11 patients with 2 *parkin *mutations and 11 sex-matched patients with idiopathic PD found the prevalence of RLS to be 45% and 0% respectively (*p *= 0.04), providing stronger evidence of an association [39].

However, other genetic evidence has not yielded any conclusive evidence of a link between RLS and PD. There is a single report of a PD patient with a *PINK1 *mutation presenting with RLS symptoms [40]. A family with hereditary PD, essential tremor and RLS was also recently described. Of 65 family members examined, 11 had PD and 15 had RLS (of which 3 had both PD and RLS) with an autosomal dominant inheritance pattern. However, testing for known gene mutations in PD -associated genes was negative [41]. A study investigating a possible genetic association of the COMT val158met polymorphism with RLS yielded negative results. The COMT val158met polymorphism, which leads to reduced COMT activity, is linked to higher dopamine availability and can influence phenotypic manifestations in PD [42].

### Clinical association studies

A number of clinical studies examining the association between RLS and PD have been conducted since the introduction of IRLSSG criteria, with variable findings [43-52]. In a study of 126 PD patients and 128 healthy age-and sex-matched controls in India, RLS was found to be present in 10 patients (7.9%) and 1 control (0.8%, *p *= 0.01) [43]. PD patients with RLS were older and had a higher prevalence of depression than those without RLS.

Ondo et al [44] evaluated 303 PD patients and found 63 (20.8%) had symptoms of RLS. Neither PD patient demographics nor PD treatments could reliably predict the development of RLS symptoms. In 54 (68%) of 79 patients with PD/RLS, the PD symptoms preceded the RLS symptoms. Compared with patients with idiopathic RLS, patients with PD/RLS were older at RLS onset, were less likely to have a family history of RLS, and had lower serum ferritin levels.

A study in the Japanese population also found prevalence of RLS to be significantly higher in PD patients than in control subjects (12% vs. 2.3%) [45]. However, PD patients with RLS were younger than those without RLS. A cohort of 113 PD patients from Austria, revealed that the 24% with comorbid definite RLS were younger (63.1+/-8.6 vs. 68.8 +/-9.0 years; *p *= 0.004), and received lower levodopa equivalent doses [46].

A Brazilian study reported the prevalence of RLS to be 18.75% in 48 patients with PD. No significant differences were observed regarding clinical variables or biochemical parameters [47]. It has also been suggested that long-term antiparkinson therapy rather than PD itself contributes to the development of RLS. A Korean study found prevalence of RLS among 447 PD patients to be 16.3%, with multivariate logistic regression analysis revealing duration of antiparkinson treatment to be the most significant factor contributing to the development of RLS in this group [48].

A few studies have commented of findings not supportive of co-morbid occurrence of PD and RLS. A case control survey of the prevalence of definite RLS in 118 PD patients did not demonstrate a significantly increase prevalence of either primary RLS alone or combined with secondary RLS as compared to gender and age matched controls [49].

Of 269 Caucasian PD patients studied in the Netherlands, 11% had definite RLS, similar to the prevalence of RLS in the general population. PD-RLS patients were more likely to be female (69% vs. 32%, *p *< 0.001), although this was the only significant difference between the 2 groups. Severity of RLS symptoms correlated positively with PD severity. Both studies acknowledged that the prevalence of RLS may have been underestimated due to dopaminergic treatment [50].

Tan et al [51] studied 125 PD patients in Singapore and found that 19 (15.2%) patients had motor restlessness. Of these, one (0.8%) patient had RLS-like symptoms closely correlated to wearing "off" effect of levodopa. However, none of the patients satisfied the IRLSSG diagnostic criteria of RLS. This was not significantly different compared to the 0.6% and 0.1% RLS prevalence in the general population and clinic population.

A subsequent case-control study of 400 study subjects in Singapore demonstrated only a weak association between RLS and PD, with prevalence of RLS in PD patients and controls 3.0% and 0.5% respectively (*p *= 0.07) [52].

### Functional imaging studies

Functional imaging studies have demonstrated that there is decreased [18 F]-dopa uptake and [123I]-ß-CIT binding in PD. However, the findings in RLS have been inconclusive, with some studies showing mild reduction in postsynaptic dopaminergic status [53,54], and another, normal D2 receptor binding compared to controls [55]. Two [18 F]-dopa PET studies demonstrated a slight decrease in striatal [18 F]-dopa uptake in RLS patients compared to healthy controls [54,56], suggesting presynaptic dopaminergic dysfunction in the striatum. However, a third study showed normal values for presynaptic dopaminergic function [57].

### Sonographic studies

Sonography of 41 patients with idiopathic RLS, 19 with RLS-PD, 25 with idiopathic PD and 35 controls revealed a significant decrease in substantia nigra (SN) region echogenicity in the idiopathic RLS group compared with other groups (*p *< 0.0001). In contrast, the PD-RLS group had increased echogenicity of the SN area compared with the control group (*p *< 0.05) and idiopathic RLS group (*p *< 0.0001), suggesting different pathological processes underlying idiopathic and PD associated RLS [58]. Another sonographic study of 63 PD patients of which 26 had comorbid RLS, 40 patients with idiopathic RLS and 40 controls demonstrated similar findings [59].

### What impact does DBS surgery for PD have on RLS?

The emergence of RLS after subthalamic nucleus (STN) deep brain stimulation (DBS) in patients with PD has been reported [60]. Eleven of 195 patients with STN DBS reported new problematic symptoms of RLS after surgery. The mean reduction in antiparkinsonian medication was 74%. The authors suggested that reduction of anti-parkinsonian medication during STN DBS may unmask symptoms of RLS. However, a recent prospective study of 17 patients undergoing STN DBS identified 6 patients with RLS with a mean IRLSSG rating score of 23 preoperatively. Postoperative scores at 4 weeks and 6 months were significantly improved at 14.8 (*p *= 0.027) and 13.8 (*p *= 0.037) respectively. None of the patients developed RLS postoperatively [61].

### Problems with the common pathophysiology theory

Symptoms of RLS in PD are milder than in patients with idiopathic disease [44] and may be difficult to differentiate from other sensory and motor symptoms in PD, in particular akathisia affecting the lower extremities [62,63]. RLS symptoms may also be a manifestation of wearing-"off" phenomenon, a levodopa related complication of PD [51,64].

Prolonged dopaminergic therapy in RLS patients, in particular with levodopa use, may result in a phenomenon known as augmentation in which the severity of symptoms increases, onset of symptoms begin earlier in the day and more rapidly, and spread of distribution to other body parts occurs. In contrast, PD patients develop dyskinesias and motor fluctuations after treatment with dopaminergic agents. These complications are not seen in RLS patients.

Total iron and ferritin levels in the substantia nigra region are increased in PD, causing oxidative stress and possibly leading to dopaminergic degeneration [65]. On the other hand, RLS is a disease of iron deficiency [66-69]. CSF ferritin levels are low and CSF transferrin levels are high in RLS patients compared to controls [70]. RLS severity negatively correlates with serum ferritin levels [69], even when the latter are within the normal range, and symptoms improve with replacement of iron [21]. Utilizing MRI techniques, one study examined regional brain iron concentrations in RLS and controls [71]. Iron was significantly decreased in the substantia nigra, and somewhat less significantly in the putamen, both in proportion to RLS severity suggesting that brain iron insufficiency may occur in some RLS patients. In a transcranial ultrasound study, RLS patients had significantly reduced midbrain areas of hyper-echogenicity compared with control subjects, and even more markedly reduced hyper-echogenicity compared with PD [72]. A neuropathological study demonstrated increased transferrin with decreased transferrin receptor expression in neuromelanin cells from the substantia nigra of four RLS brains compared to four control brains. The findings were consistent with iron deficiency except that transferrin receptor expression was decreased rather than increased. Decreased iron regulatory protein 1, involved in the post-transcriptional regulatory mechanism for transferrin receptor expression, was found in the RLS brains. The authors postulated that RLS may result from decreased iron regulatory protein 1 in neuromelanin cells resulting in destabilization of the transferrin receptor mRNA and therefore cellular iron deficiency [73].

Hypofunction of the endogenous opioid system has been postulated to be a mechanism related to the pathogenesis of RLS. Exposure to the iron chelator desferoxamine in cell cultures of rat substantia nigra resulted in DNA fragmentation while pre- administration of enkephalin (a delta opioid peptide) significantly protected the cells from damage by iron deficiency [74].

### Future prospects: questions to be answered

The link between PD and RLS has yet to be clearly determined with clinical association studies differing widely in their findings, with some finding the incidence of RLS to be much greater in PD patients, and others finding no difference from that in the general population. These discrepancies could be addressed with prospective long-term clinical studies of PD patients who develop RLS and vice versa, with documentation of exposure to dopaminergic therapies.

With the exception of the parkin mutation, genetic studies have been failed to reveal any associations. We propose that population based genetic association studies of PD plus RLS and linkage studies of PD plus RLS as well as comparative studies of PD vs. PD-RLS vs. RLS should be conducted.

Sonographic studies reveal notable differences between PD and PD-RLS patients. Functional MRI studies have yet to focus on those patients with PD-RLS. Prospective functional imaging studies of PD vs. PD-RLS are needed to better understand the mechanisms involved in these disorders. To our knowledge, there have been no pathological studies looking at patients with PD-RLS. Establishing the pattern of decreased D2 receptor density in PD vs. PD and RLS vs. RLS may be valuable in understanding common pathophysiology. The reports purporting to DBS and RLS are conflicting, and more studies need to be done to clarify the effect, with attention to adjustments in dopaminergic medications.

## Conclusions

Certain similarities and differences exist between RLS and PD. Dopaminergic disturbance appears to underpin both diseases suggesting that there may be some overlap in pathophysiology. However, there is sonographic evidence that the actual mechanisms may not be identical. A recent study suggests that the nigrostriatal dopaminergic system, primarily involved in PD, is also affected in RLS. The literature appears to support a link between the parkin mutation and RLS, although other genetic studies have yielded mostly negative results. While some advances have been made in understanding the link between RLS and PD, much is yet to be elucidated particularly with regard to pathologic mechanisms and the role of genetics. Prospective long-term clinical studies are indicated to evaluate the exact relationship between PD and RLS.

## Abbreviations

RLS: restless legs syndrome; PD: Parkinson's disease; DBS: deep brain stimulation; HD: Huntington's disease; IRLSSG: International Restless Legs Syndrome Study Group; PLMS: periodic leg movements of sleep; TS: Tourette syndrome; SNPC: substantia nigra pars compacta.

## Competing interests

The authors declare that they have no competing interests.

## Authors' contributions

TP carried out the literature search and drafted the manuscript. EKT contributed to conception and revision of the article. All authors read and approved the final manuscript.
